# New insight on the role of localisation in the electronic structure of the Si(111)(7 × 7) surfaces

**DOI:** 10.1038/s41598-021-94664-w

**Published:** 2021-07-22

**Authors:** M. E. Dávila, J. Ávila, I. R. Colambo, D. B. Putungan, D. P. Woodruff, M. C. Asensio

**Affiliations:** 1 Materials Science Institute of Madrid (ICMM), Spanish Scientific Research Council (CSIC), 28049 Cantoblanco, Madrid Spain; 2 Synchrotron SOLEIL, L’Orme des Merisiers, Saint Aubin-BP 48, 91192 Gif sur Yvette Cedex, France; 3 Institute of Mathematical Sciences and Physics, University of the Philippines Los Baños, 4031 Los Baños, Laguna Philippines; 4 Physics Department, University of Warwick, Coventry, CV4 7AL UK; 5 MATINEE: CSIC Research Associated Unit Between the Institute of Materials Science of the Valencia University (ICMUV) and the ICMM, 28049 Cantoblanco, Madrid Spain

**Keywords:** Electronic properties and materials, Two-dimensional materials, Nanoscale devices

## Abstract

New angle-resolved photoelectron spectroscopy (ARPES) data, recorded at several different photon energies from the Si(111)(7 × 7) surface, show that the well-known S1 and S2 surface states that lie in the bulk band gap are localised at specific (adatom and rest atom) sites on the reconstructed surface. The variations in the photoemission intensity from these states as a function of polar and azimuthal emission angle, and incident photon energy, are not consistent with Fermi surface mapping but are well-described by calculations of the multiple elastic scattering in the final state. This localisation of the most shallowly bound S1 state is consistent with the lack of significant dispersion, with no evidence of Fermi surface crossing, implying that the surface is not, as has been previously proposed, metallic in character. Our findings highlight the importance of final state scattering in interpreting ARPES data, an aspect that is routinely ignored and can lead to misleading conclusions.

## Introduction

The continuing push for higher and higher degrees of integration in electronic devices, together with the emergence of novel two-dimensional (2D) layer materials such as silicene and graphene, are driving an increasing need to understand the electronic structure of materials confined to a thickness of only a few atomic layers^1^. Angle-resolved photoelectron spectroscopy (ARPES) has proved to be particularly successful in determining directly the electronic band structures and momentum-resolved electronic self-energies of a wide range of materials including many 2D quantum materials^2–4^, high Tc cuprates and iron-based superconducting cuprates^5,6^, and a wide variety of topological state of matter^7,8^. Indeed, ARPES is a particularly effective probe of the band structure of 2D materials, including states trapped at solid–vacuum interfaces; electrons in these states are itinerant within the 2D material but localised perpendicular to this plane, so the energy bands are determined only by the electron momentum within the layer, *k*_*//*_, a quantity that is conserved during the photoemission process. Peaks in the photoemission spectra at specific emission angles can therefore be related directly to the *E-k*_*//*_ values of the initial electronic band states. Moreover, a map of the intensity of photoemission from occupied states at Fermi level as a function of *k*_*//*_ provides a direct representation of the 2D Fermi surface of the investigated materials^9^.

ARPES has been used in a number of studies to investigate the properties of the two surface states present on the (7 × 7) reconstructed Si(111) surface, which are generally believed to be 2D itinerant band states with a characteristic 2D Fermi surface being mapped by this technique. Here we present new ARPES data from this system, recorded at several different photon energies, that call this interpretation into question. Specifically, the intensity variations of the surface state emission recorded in ARPES is shown to be dominated by final-state photoelectron scattering consistent with localised emission from two distinctly different atomic sites within the reconstructed surface. This severely distorts the photoemission intensity map in *k*_*//*_ that would normally be thought to show the 2D Fermi surface. As is well known, ARPES from atomic core levels, which are fully 3D localised, is dominated by this final state scattering, namely the interference of the outgoing photoelectron wave and elastically-scattered components of this wave from surrounding atoms, This leads to a well-established structural technique of photoelectron diffraction^10–12^ the scattering interference being simulated by multiple scattering calculations (MSC), to allow a quantitative determination of the emitter's local structural environment. Previous studies of ARPES from itinerant band states in metals^12^ and cuprate superconductors^13^ have demonstrated the importance of this final state scattering, but the results presented here highlight how neglecting this effect can lead to misleading conclusions regarding the electronic structure.

**Figure 1:**
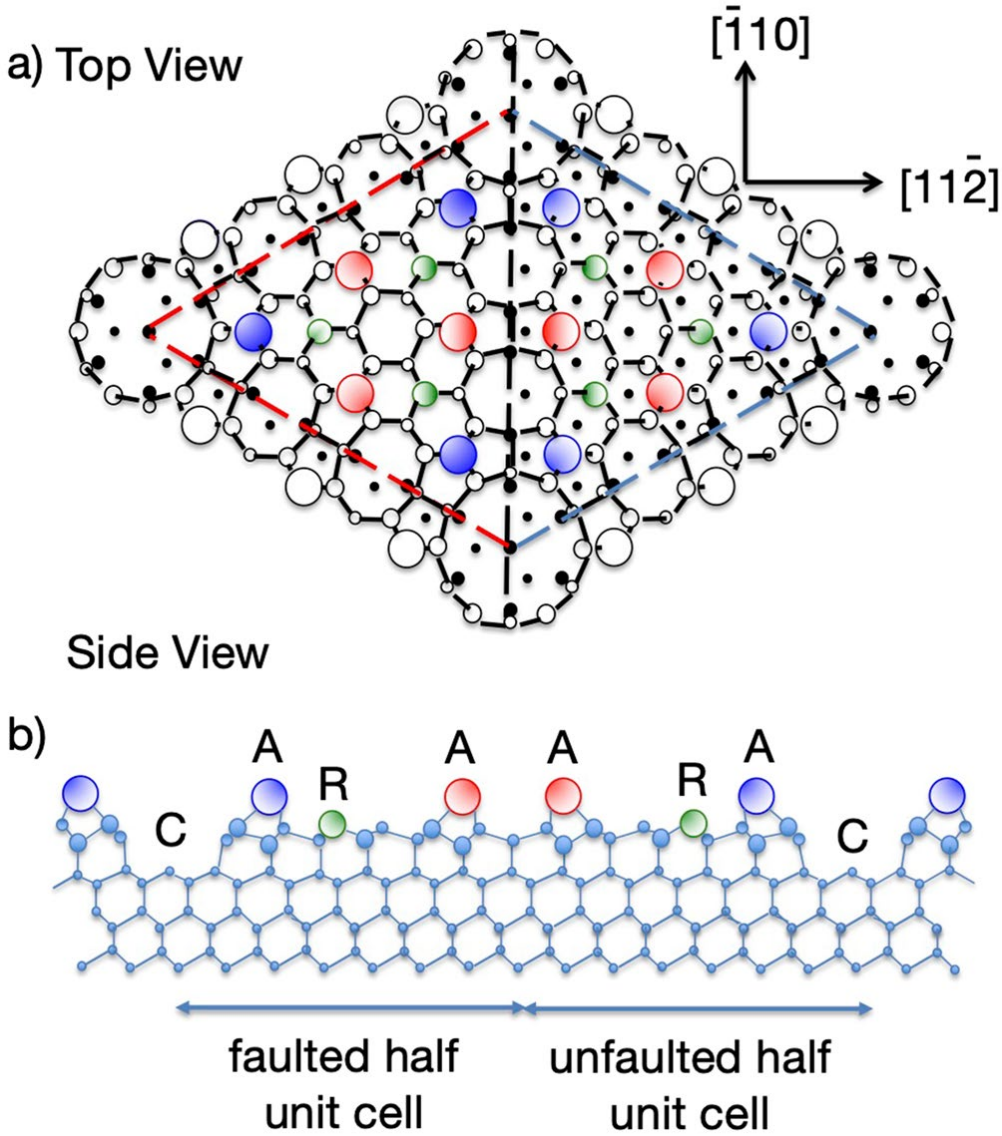
Schematic diagram of the DAS model of the Si(111)(7 × 7) surface showing the adatoms (A), rest atoms (R) and the corner rest atoms (C). The top view shows the different visibility of lower atoms in the two halves of the unit mesh due to the presence of a stacking fault in one half.

Despite extensive investigation of the 2D electronic structure and conductive properties of the surface of Si(111), in its stable (7 × 7) reconstruction, key aspects remain controversial. The surface reconstruction is well-known to lead to the emergence of two surface localised states, generally labelled S1 and S2, in the bulk band gap of Si, at binding energies relative to the Fermi level of ~ 0.2 eV and ~ 0.8 eV, respectively, although there is evidence that the S1 state is split into 2 or 3 distinct components (e.g.^14,15^). These states are generally believed to form 2D itinerant band states and have been extensively studied by ARPES^14–18^. The widely-accepted structural model of the Si(111)(7 × 7) surface reconstruction is the so-called dimer-adatom-stacking fault (DAS) structural model first proposed by Tagayanaki et al.^19^ although very recently alternative polymorphs have been proposed^20,21^. Within this large unit mesh the number of Si dangling bonds produced by cleavage of the bulk crystal is reduced from 49 to 19 by the associated reconstruction of the outermost Si layers, significantly lowering the surface energy. These remaining dangling bonds are on Si adatoms, bonded to three underlying ‘bulk’ Si atoms, and on ‘rest’ atoms in the lower layer that retain their original dangling bond character. Figure 1 shows the DAS structural model, identifying the local geometry of the adatoms and rest atoms. Charge rearrangement is believed to occur with the six rest atoms and the corner hole rest atom, each accepting one electron from an adatom, leaving the 12 adatoms in the outermost layer with only 5 electrons. This odd number of electrons implies that the surface should be metallic.

Several measurements of the surface conductivity have been reported^22–24^ but are somewhat inconclusive because of the influence of mobile carriers in bulk. Standard Density Functional Theory (DFT) calculations do support the metallic surface picture, reporting a band attributable to the experimentally observed S1 photoemission peak crossing the Fermi level^15,25^, and while calculations taking account of strong correlation effects^26,27^ paint a somewhat different picture with some localisation (supported by some β-NMR results^28^, they also predict a metallic surface. The results of several ARPES experimental studies, however, provide some conflicting evidence as to whether the band does, or does not, cross the Fermi level. One complication in interpreting these data is that in room temperature measurements the S1 peak is rather broad, but while making the measurements at low temperature (down to ~ 20 K or less) leads to much sharper peaks, this does introduce a significant surface photovoltage shift that must be corrected for to determine the true binding energies relative to the Fermi level. Of the two studies at the lowest temperatures, one concludes that the band does cross the Fermi level^17^, the other that it does not^15^. Of the experiments performed at higher temperatures, there seems to be an assumption that Fermi level crossing occurs, leading of the photoemission intensity from the S1 state as a function of k// being interpreted as 2D Fermi surface maps, although E-k// plots mostly show very weak dispersion with no explicit evidence of EF crossing.

In contrast to these studies implicitly assuming that the S1 and S2 surface states correspond to 2D itinerant band states, scanning tunnelling spectroscopy (STS) measurements indicate that the S1 and S2 states are localised at the Si adatoms and Si rest atoms, respectively^23,29^ which would seem to imply the surface is not metallic, although the STS results could be influenced by the proximal and localised character of this type of nano-probe. Despite this vigorous debate, published ARPES data from these two surface states have been exclusively interpreted in terms of the momentum (k)-space initial state dispersion, disregarding the localisation evidence.

Here we present clear evidence that, contrary to this conventional wisdom, ARPES from the weakest bound surface states of the Si(111)(7 × 7) surface is not dominated by the effects of k// dispersion in the initial state, but by final state scattering. Our measurements of the binding energy dispersion (E vs. k//) and full surface state photoemission angular intensity patterns, recorded using different incident photon energies, clearly show that their intensity modulations cannot be interpreted in terms of band mapping. Instead of, we show that final-state scattering of local surface states emitters dominates the angular distribution of these photoemission intensities and that these patterns can be reproduced by MSC from specific emission sites associated to distinctive atoms in the Si(111)7 × 7 surface reconstruction. Consequently, the interpretation of the angular dependence of the photoemission intensity in terms of a band/Fermi surface mapping picture is inappropriate. The interpretation of these data in a band picture and its implications for surface conductivity is therefore potentially very misleading. The ARPES maps of the S1 state cannot be taken as a 2D Fermi surface plot.

**Figure 2:**
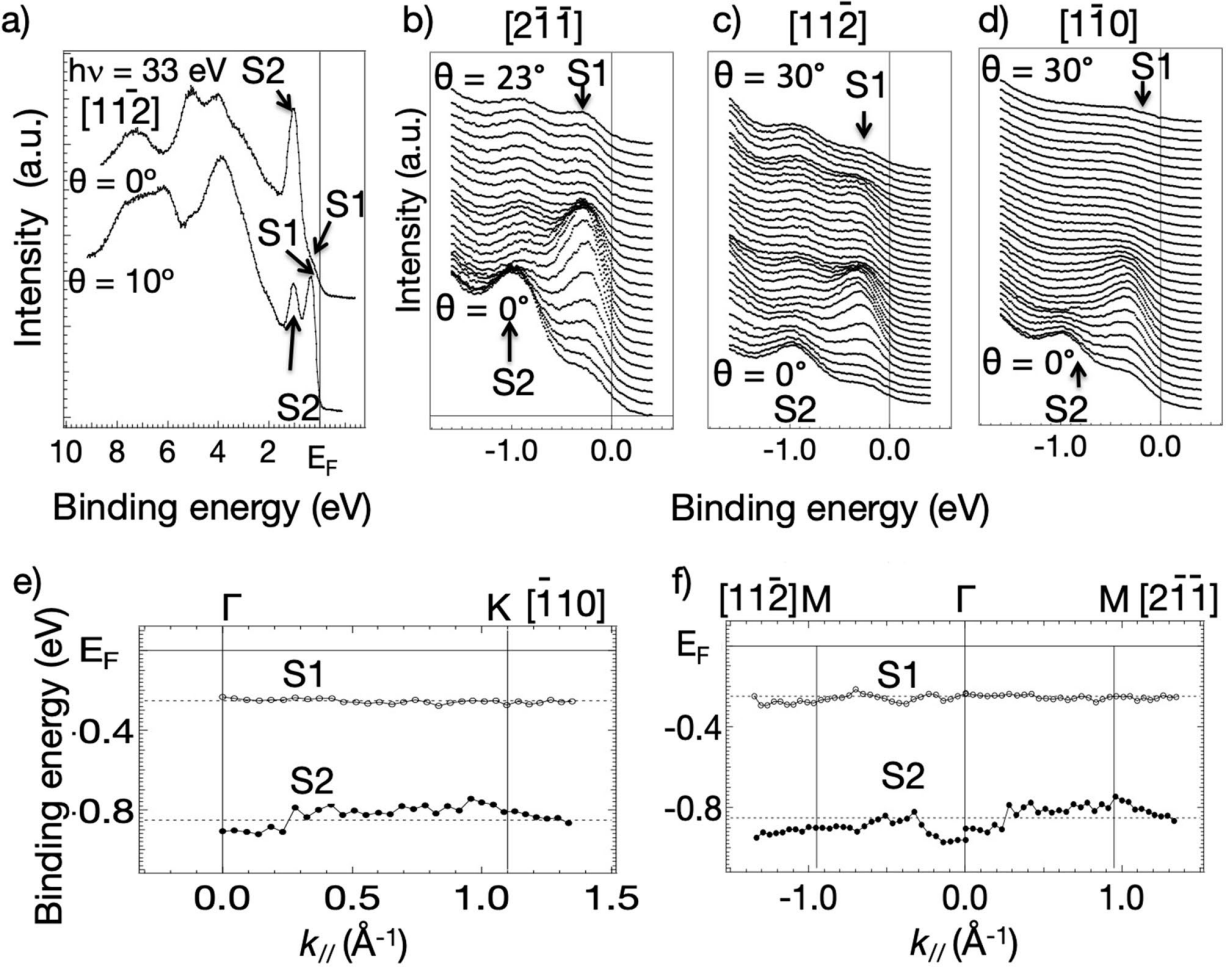
(**a**) ARPES spectra recorded at 33 eV of the valence region of Si(111)(7 × 7) at normal emission (θ = 0°) and at a polar angle θ = 10° in $[11\overline{2} ]$ azimuth. (**b**–**d**) ARPES of the energy range of the S1 and S2 states at a sequence of different polar emission angles in each of the three principal azimuthal directions. (**e**,**f**) mapping of the S1 and S2 initial state energies as a function of k//, in the two principal azimuths. See Figure S1 for further details. The data have been measured using setup I, described in Figure S2 (Figures S1 and S2 described in supplementary information).

**Figure 3:**
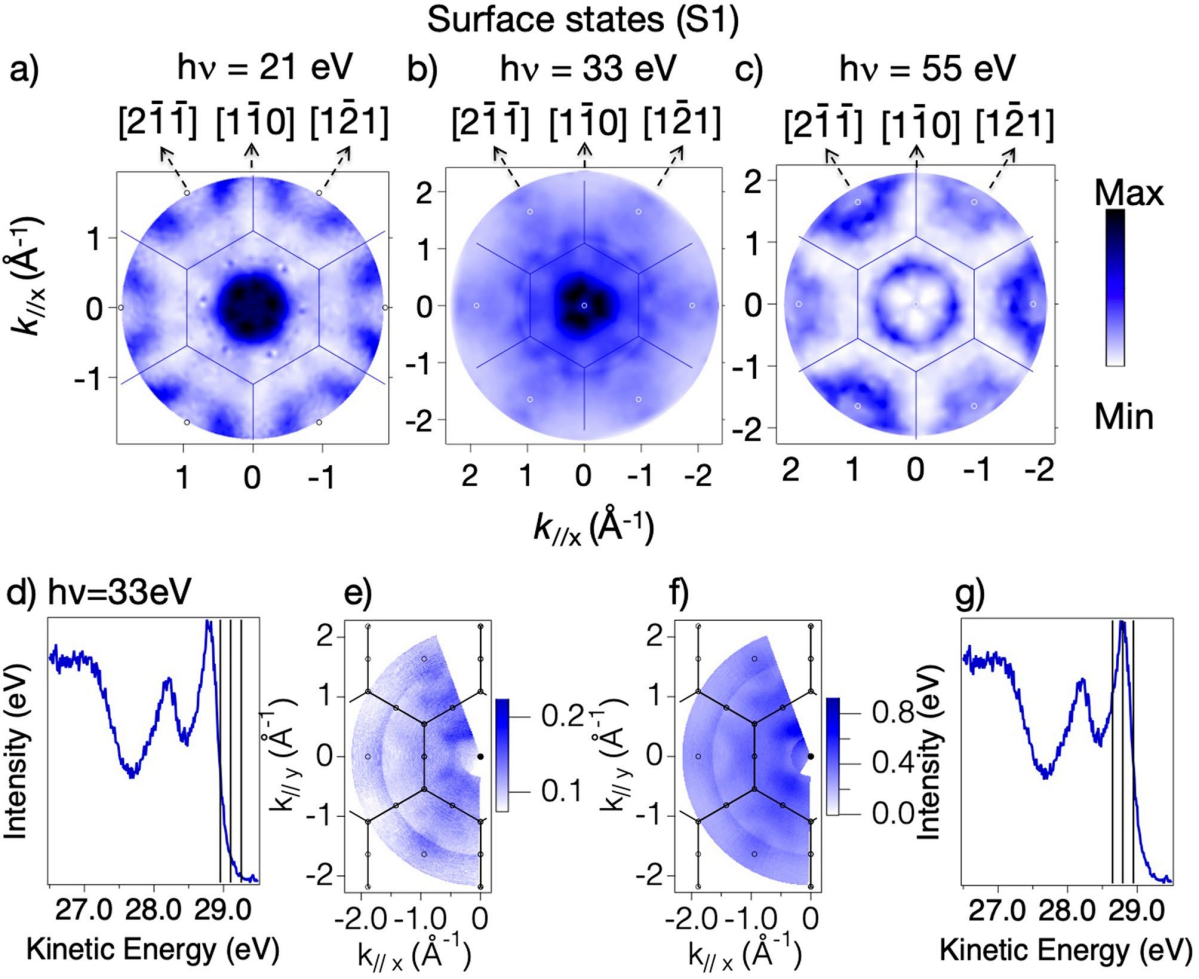
(**a**–**c**) for map of the intensity of the photoemission from the S1 state as a function of k// recorded at three different photon energies (21 eV, 33 eV and 55 eV respectively). The superimposed lines correspond to the (1 × 1) Brillouin zone boundaries. (**e**,**f**) compare similar partial maps recorded at 33 eV from emission from the S1 peak (**g**) and from the Fermi level (**d**). Panels (**a**–**c**) have been recorded using the setup I of Figure S2, while panels (**d**–**g**) have been measured in the setup II of Figure S2.

Figure 2 shows a series of ARPES spectra recorded at 33 eV from the valence states of the Si(111)(7 × 7) surface as a function of emission angle, together with mapping of the angle-dependence of the initial state energies of the S1 and S2 peaks as a function of the electron momentum parallel to the surface. These data show very little variation of the binding energy with k// and thus very little evidence of dispersion The S1 state binding energy appears to always be below the Fermi level. However, if this state does straggle the Fermi level, as proposed in several ARPES studies^17,18^ a kx-ky map of the intensity of this peak as a function of the initial state k// value should correspond to a map of the 2D Fermi surface. Maps of this type obtained from our data are shown in Fig. 3a–c for the S1 state recorded at three different photon energies (21 eV, 33 eV and 55 eV respectively). If the intensity variations shown in these three maps were dominated by initial state k// conservation effects one would expect them to be similar, qualitatively independent of the photon energy, because for 2D states the Fermi surface depends only on k//. (This contrasts with ARPES from 3D itinerant states in which different photon energies access different values of the perpendicular component of the electron momentum, k⊥, leading to different cuts of the 3D Fermi surface). Clearly this is not the case; there are pronounced differences between maps of the same initial state, recorded with different photon energies corresponding to different final states. Of course, it is also true that if S1 does not cross the Fermi level these maps should not show the 2D Fermi surface. However, as it can be seen in panels (d-g) of Fig. 3, the maps recorded at 33 eV for the S1 peak intensity (Fig. 3f) and for emission from the ‘tail’ of the S1 peak at the Fermi level (Fig. 3e) are essentially identical.

**Figure 4:**
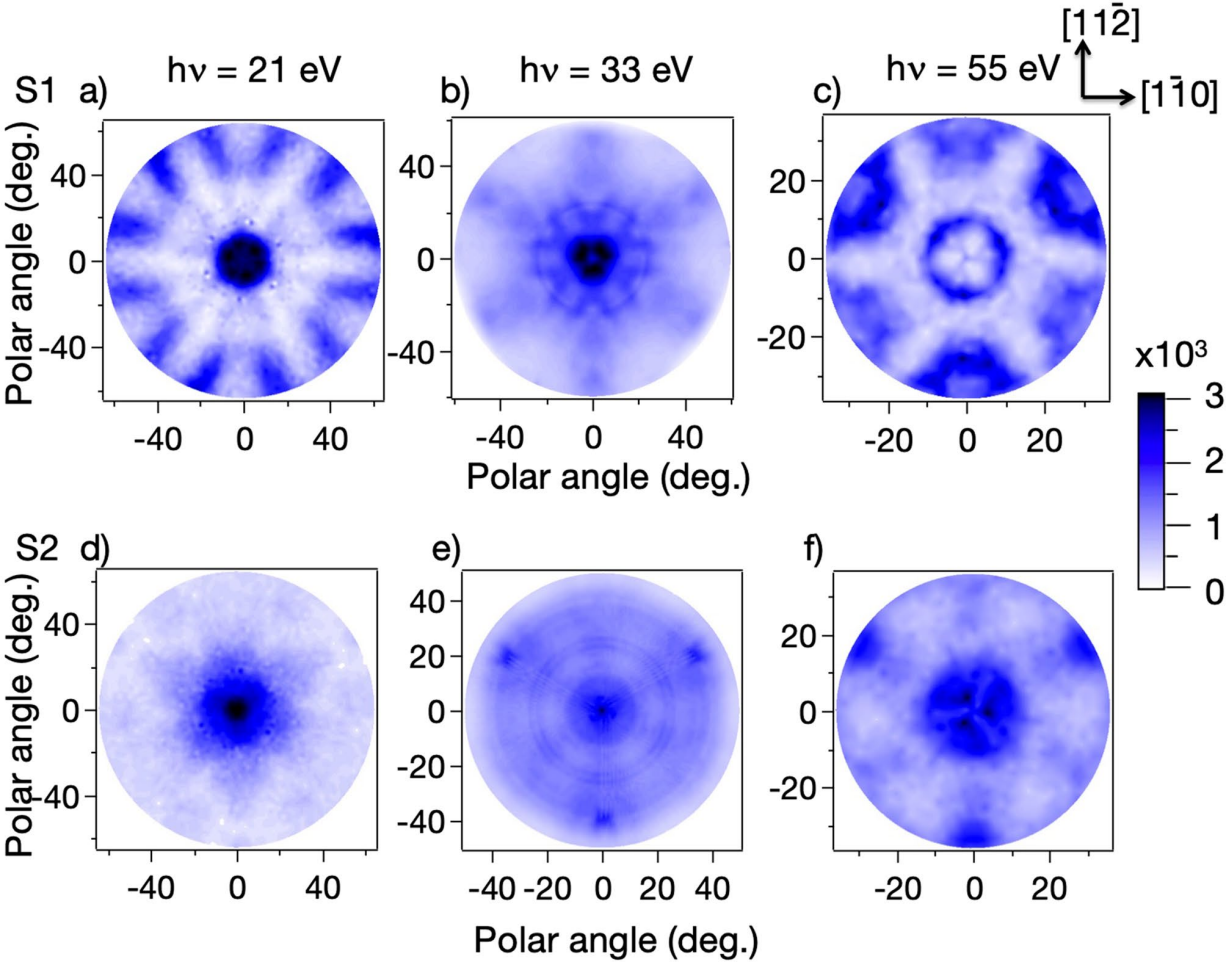
Stereographic projections of the intensity of photoemission from the S1 represented in panels (**a**–**c**) and S2 in panels (**d**–**f**) surface states on Si(111)(7 × 7) at three different photon energies (21 eV, 33 eV and 55 eV respectively). All the holograms have been recorded using the setup I of Figure S2.

Given the evident importance of the final state energy, despite the binding energy of this surface state being very low, we have explored the impact of an entirely different mechanism that may determine these different ARPES patterns. Specifically, we assume that the S1 and S2 states are not 2D itinerant band states but are local to the adatoms and rest atoms, respectively. In that case, we can use the standard MSC codes describing elastic scattering in the final state to determine the photoelectron diffraction angular patterns from these two local sites, in exactly the same fashion as is exploited in photoelectron diffraction from core levels. For this purpose, it is now appropriate to plot the angular dependence of the S1 and S2 photoemission intensities not as a function of their k// values, but rather as a function of the polar and azimuthal emission angles. This can be done using a stereographic projection, and the data of Fig. 3 are shown in this format in Fig. 4 for S1 and also for S2. These intensity patterns clearly show that the angular distributions from the S1 and S2 states are quite different and depend strongly on the photon (and thus the photoelectron) energy. Consequently, these emission intensity patterns appear to be dominated by final state scattering and not by initial state k// conservation effects. As such, they do not map the 2D Fermi surface of Si (111)7 × 7.

**Figure 5:**
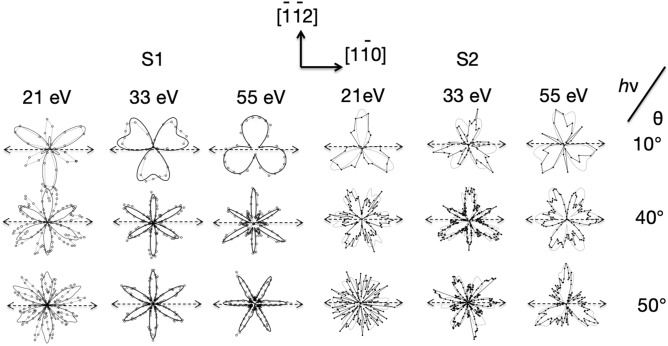
Normalised polar diagrams (at 10**°**, 40**°** and 50**°** polar angles) of the variation of photoemission intensity from the S1 and S2 surfaces state, obtained from the spectrum of photoemission intensity for a defined polar angle minus the minimum intensity value of this spectrum and divided by the difference between the maximum and minimum intensity values of this spectrum (Inormalised = (Iϕ − Imin)/(Imax − Imin)), as a function of azimuthal angle at several different photon energies (21 eV, 33 eV and 55 eV) and polar emission angles (at 10**°**, 40**°** and 50**°** polar angles). The experimental data, shown as individual data points joined by straight lines, are compared with the results of the MSC calculations shown as continuous curves.

A more quantitative comparison between the experimental and computed angular distributions can be achieved by plotting the variations in emitted intensity as a function of azimuthal angle, ϕ, at a series of fixed polar angles. The theoretical MSC intensity modulation curves have been calculated on the assumption that the emission from the S1 and S2 states are localised at the sites of the adatoms and rest atoms, respectively, taking their positions from the well-known DAS structural model. The results of these comparisons are shown in Fig. 5 for both the S1 and S2 states, as polar plots (at 10°, 40° and 50° polar angles) of the normalized modulations, (Iϕ − Imin)/(Imax − Imin) as a function of azimuthal emission angle at different photon energies (21 eV, 33 eV and 55 eV), where Imax and Imin, are the maximum and the minimum intensity values of the S1 peak a given polar emission angle.

In general, the agreement between the experimental and computed photoelectron diffraction azimuthal patterns is good; the results of the MSC calculations clearly reproduce all the main trends as a function of energy and emission angle, although some more detailed aspects are less well reproduced. Specifically, the calculations reproduce the strong azimuthal intensity modulation of the emission from the surface states as the photon energy is varied, but also reproduce the difference in the patterns from the S1 and S2 states when their emission is calculated to be from adatom and rest atom sites, respectively. This evidence conflicts with the previous interpretation of such data that the intensity variations relate to the Fermi level crossing due to the dispersion of an itinerant 2D band state^15,17,18^.

While our MSC simulations that provide an excellent description of the observed ARPES patterns assume that emission from the S1 and S2 states is from the atomic sites of the adatoms and rest atoms, this does not imply that these surface states are fully 3D-localised at these atoms. As has been shown previously^12^, similar MSC calculations of the final state scattering match the ARPES from bulk band states (notably the 3d band of Cu) when the experimental resolution averages over all or much of the band. This effect can be attributed to the photoemission matrix element, most clearly in its ‘acceleration’ form, in which the emission is shown to be dominated from regions where the electron potential has its steepest gradient—i.e. at the atomic cores. However, unlike these earlier ARPES studies of bulk states in an elemental solid, in which all atomic sites are equivalent, in the Si(111)7 × 7 surface the adatom and rest atom sites are structurally distinct, and our results show that the S1 states must be formed from local orbitals centred on the adatoms, whereas the S2 states are composed of atomic orbitals centred on the rest atoms.

This conclusion is, of course, consistent with that of previous STS studies which also indicated that the S1 and S2 surface states on this surface are localised at the adatom and rest atom sites, respectively, but subsequent ARPES studies have, instead, assumed that these are 2D itinerant band states. Our results show the ARPES data from the Si(111)(7 × 7) surface are not consistent with this interpretation. ARPES data recorded from a wide range of 2D materials and surfaces, including the surfaces of metals, are analysed in terms of the 2D band picture. In the great majority of these, there is no reason to doubt that this is not correct. Nevertheless, our results highlight a need for caution in interpreting such data, and the potential importance of final state scattering effects, particularly if recorded from states that show little evidence of dispersion in k//, including flat bands close to the Fermi level.

In conclusion, we show that elastic scattering in the final state dominates the intensity modulation patterns from the two surface states on Si(111)(7 × 7). Indeed, we reveal that both S1 and S2 intensity modulations of the (7 × 7) reconstruction surface states close to the Fermi level can be fully reproduced by detailed multiple scattering calculations, assuming that these surface states are localised at the adatom and rest atom sites, respectively. Our findings confirm that the strong intensity modulation features of the S1 emission from this surface, previously associated with surface states k// dispersion crossing the Fermi level (i.e., previously believed to map the 2D Fermi surface), are due to photoelectron diffraction interference effects of the outgoing photoelectron wavefield. Our results do not exclude the possibility that these surface states may form 2D bands due to hybridisation of the local orbitals on these specific surface atoms, but the lack of significant dispersion is consistent with weak hybridisation. As both states lie below the Fermi level the clear implication is that the surface is not metallic. Interestingly, there is a large body of experimental and theoretical evidence indicating that many of the structural building blocks of the DAS reconstruction are also present in other surfaces, interfaces, and few-layer structures of a wide variety of semiconductors^30–33^. Indeed, similar structural building blocks have also recently been reported for silicene^34,35^, suggesting that DAS-associated localised electronic states may also be present in other types of silicon nanostructure.

## Methods

### Experimental set-up

All data were recorded from silicon (111) wafer samples, cleaned only annealing in ultra-high vacuum (UHV) at 950 °C and short flashes up to 1050 ºC, with no ion beam bombardment. The UHV system for the experiments was equipped with a four-grid LEED optic and a quadrupole mass spectrometer. The temperature of the sample could be varied between 100 and 1000 K by liquid N2 cooling and resistive heating through tungsten wires which held the crystal in position. The temperature of the sample was measured by a thermocouple in direct contact with the crystal. Notice that the S1 and S2 photoemission peaks are extremely sensitive to contamination, providing a valuable monitor of the state of surface cleanness.

### Angle-resolved photoemission spectroscopy (ARPES)

ARPES measurements were performed first at the ANTARES beamline installed at LURE (ARPES set-up I of Figure S2) and subsequently at the renovated same beamline at SOLEIL synchrotron, using an ARPES set-up type II of Figure S2, in Orsay and Gif-sur-Yvette, France, respectively. All data were acquired using a light beam of linearly polarized photons with a 120 μm spot size, tuning the incident photon energy in the range of 21 eV to 55 eV. The photoemission angular distribution maps themselves represent the total photoelectron intensity within a narrow region of energy and angle, scanning a large set of emission angles and recording the photoelectron intensity as a function of kinetic (and thereby initial state binding) energy. The photoelectron spectra were obtained using a fixed concentric hemispherical analyser (Scienta R4000) at Soleil, and a movable small hemispherical analyser at LURE, with energy and momentum resolution of 5 meV and 0.005 A − 1, respectively. The Scienta detector was aligned in the plane of the synchrotron ring and the acceptance angle of the lens could be set either to 1°or 25° for angular mappings. The photoemission measurements at LURE and ANTARES were performed with the sample at room temperature.

### Multiple scattering cluster calculations

Quantitative determination of the structure associated with the local scattering around the adatoms and rest-atoms from the measured photoelectron diffraction spectra requires the use of extensive multiple-scattering cluster calculations. Within a first-order perturbation theory the intensity of the outgoing photoelectron can be calculated using the Green’s function of the total system, which can be expanded into a series over all possible pathways which connect the emitter via scattering centres to the detector. The scattering simulations presented here were performed using the approach based on a magnetic quantum number expansion^36^. Further details of the MSC computational procedure presented in this work are given in the Sect. 2 of the Supplementary Information file and in the Supplemental References.

## Supplementary Information

Supplementary Information.
