# Supplementary material for: New insight on the role of localisation in the electronic structure of the Si(111)(7 × 7) surfaces
Source: Sci Rep. 2021 Jul 22;11:15034. doi: 10.1038/s41598-021-94664-w (PMC8298386; doi:10.1038/s41598-021-94664-w)
Supplement: Supplementary file 1 — Supplementary Information. [file 41598_2021_94664_MOESM1_ESM.pdf]

Supplementary information

New insight on the role of localisation in the electronic structure of the Si (111) (7x7) surfaces

M. E. Dávila[1], J. Ávila[2], I. R. Colambo[3], D. B. Putungan[3], D. P. Woodruff[4] and M. C. Asensio[1,5,*]

[1] Materials Science Institute of Madrid (ICMM), Spanish Scientific Research Council (CSIC), E-28049 Cantoblanco, Madrid, Spain.

2 Synchrotron SOLEIL, L'Orme des Merisiers, Saint Aubin-BP 48, 91192 Gif sur Yvette Cedex, France

3 Institute of Mathematical Sciences and Physics, University of the Philippines Los Baños, Laguna 4031, Philippines.

4 Physics Department, University of Warwick, Coventry CV4 7AL, United Kingdom

5 MATINEE: CSIC Research associated unit between the Institute of Materials Science of the Valencia University (ICMUV) and the ICMM, E-28049 Cantoblanco, Madrid, Spain.

CONTENT

SUPPLEMENTARY INFORMATION 1

NEW INSIGHT ON THE ROLE OF LOCALISATION IN THE ELECTRONIC STRUCTURE OF THE SI (111) (7X7) SURFACES 1

1.- Photoemission 2

1.1- Angle resolved photoemission 2

1.2- Photoemission matrix element 5

1.3- Scattering final state wave function 6

2.- Multiple scattering cluster calculations 7

2.1- Description of the Si(111)7x7 atomic cluster 7

3.- References 9

1.- Photoemission

1.1- Angle resolved photoemission

Photoemission (PES) is a well-established surface analysis technique, originally developed in conventional laboratories using monoenergetic soft X-ray (~1200-1500 eV) and vacuum ultraviolet (~20-40 eV) radiation sources, but now at large synchrotron radiation facilities, which deliver radiation tuneable over this complete energy range. The typical experimental setup for PES experiments, illustrated in Figure S1, consists of an electron spectrometer as a (movable or fixed) energy-selective detector of photoelectrons, emitted as a result of the incident photon illumination.

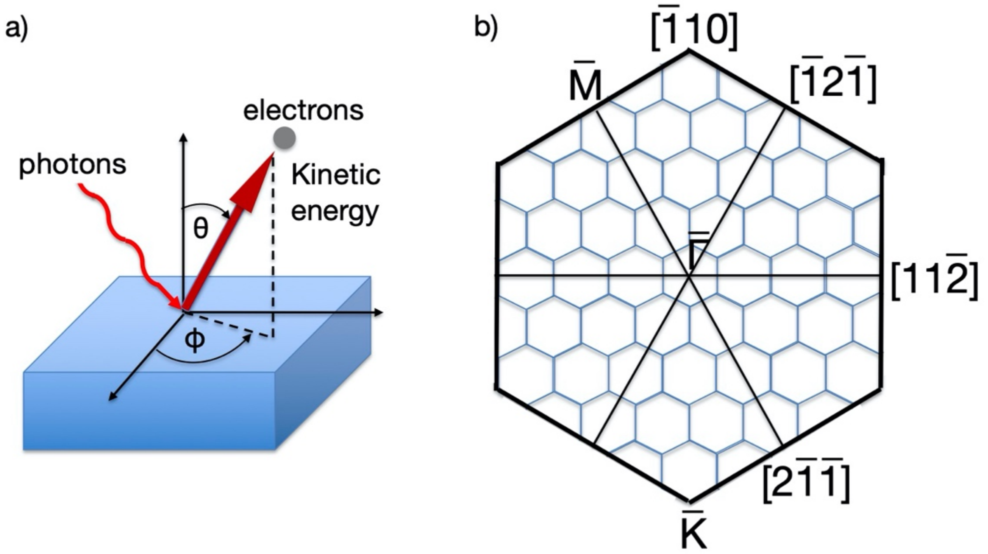

Figure S1: Panels a) and b) of the figure show, respectively, a schematic photoemission experiment and the (1x1) and (7x7) Brillouin zones of the Si(111) crystal, indicating the high symmetry points. The polar (θ) and azimuthal (ϕ) angles are referenced with respect to the normal to the surface plane. The wave vector values of the surface projected main symmetry points are: $K_{1x1}=(2\sqrt{2}/3)\ 2\pi/a=1.09\ Å^{-1}$; $M_{1x1}=(\sqrt{2}/\sqrt{3})\ 2\pi/a=0.95\ Å^{-1}$; $K_{7x7}=0.16\ Å^{-1}$; $M_{7x7}=0.14\ Å^{-1}$.

Two somewhat different modes of applications have developed. Using the soft X-ray source, PES is commonly used to study the chemical composition of surfaces, detecting core level emission at energies characteristic of the atomic species, but also detecting chemical shifts in the photoelectron binding energies. Using mostly lower photon energies, the focus is on photoemission from the valence states, proving

information on the density of states of the valence band. If these experiments are performed in an angle-resolved fashion, with good energy resolution, angle-resolved PES (ARPES) provides a precise and direct determination of the electronic band structures of materials, recording the momentum-resolved electronic self-energies straightforwardly. The conservation of the component of the electron momentum parallel to the surface, $k_{\\}$ allows the energy dispersion of the occupied valance bands to be determined as a function of the momentum, k, mapping the electronic structure (E-k dependence) throughout large portions of reciprocal space and displaying experimentally the size and symmetry of the Brillouin zone of the studied material. Figure S1 also shows Brillouin zones (BZs) of the Si (111) 1x1 and 7x7 unit cells, respectively, identifying their high symmetry directions and relative sizes.

Angle-resolved photoemission studies can also be performed on core level emission, leading to distinctly different information. Specifically, in this case the angular variations on the photoemission are determined particularly by the effect of photoelectron diffraction: the coherent interference of the directly emitted photoelectron wavefield from individual atoms with components of the same wavefield elastically scattered by surrounding atoms. The resulting interference pattern is determined by the relative position of the emitted atoms and the scattering atoms (through the scattering pathlengths), so this provides quantitative information on the local structure of the emitter atoms. Using soft X-ray illumination this technique is generally referred to as XPD (X-ray Photoelectron Diffraction), although the same effect also occurs at lower photon energies, when backscattering from the underlying substrate atoms becomes more important.

The experimental requirements to perform these two associated well-established techniques, ARPES and XPD, are essentially the same. For ARPES, good energy and momentum resolution of the analyser are essential to allow precise mapping of the electronic band structure, which may require the detection of weak energy dispersion of narrow bands, affected by small energy gaps and/or hybridisation, these features of the electronic band structure being important determinants of the properties of the investigated materials. For XPD, on the other hand, the measurements of the single energy photoelectron peaks from core levels are less demanding in resolution. For XPD the instrumental requirements are a moderate energy resolution and an effective and precise automatic motor system to vary the polar and azimuthal angles of photoelectron detection (ϕ and θ, respectively in Figure S1) in

an accurate and reproducible fashion, in order to measure the complete or partial angle-dependent intensity modulation patterns (photoelectron holograms).

In the case of XPD measurements to obtain structural information, both full intensity modulation patterns (holograms) and selective azimuthal intensity scans, obtained by rotating the sample around its surface normal (Theta angle), are measured starting from grazing up to normal emission.

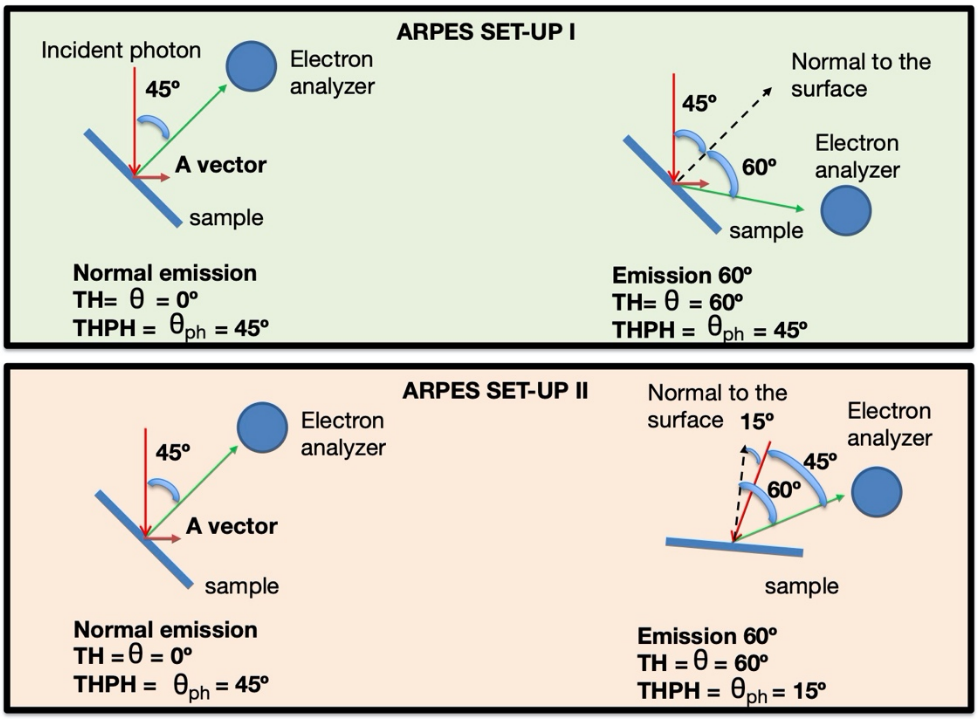

Figure S2. Two experimental setups have been used to record the hemispherical intensity modulation patterns presented in this study. Both instruments are fully equipped with a high precision two-axis sample manipulator, which allows the automatic scanning of the polar (θ) and the azimuthal (ϕ) angles, relative to the sample surface normal, of the photoelectron detection direction. For SETUP I, the analyser is mounted in the vacuum on a two-axis goniometer, allowing scanning of the polar collection angle without changing the incident photon angle. That is particularly valuable to study the effect of the polarization vector of the incident light on the hemispherical intensity modulation patterns because the incident angle of the light (θ_{ph}) and the photoelectron polar angle emission (θ) can be fixed independently. In SET-UP II, however, the analyser is fixed in the plane of the linear horizontal polarization of the light (synchrotron ring plane), defining an angle of 45º between the incident photon light direction and the outgoing photoelectron detection. In both setups, the angle-resolved experiments are performed by scanning the angles by computer-controlled stepper motors, completing the configuration with a very high precision sample platform that allows reproducibility of a hundred nanometers and an angular precision of the order of 0.1º.

The intensities are then stereographically projected as a false colour-scale map, as shown in Figures 3 and 4. The center and the outer ring correspond to normal and grazing emission, respectively; one full map contains more than 5000 angular settings. In these experiments, the sample is rotated around its two axes. The data acquisition is automated, recording the photoelectron intensities of a particular PES peak at each angular position.

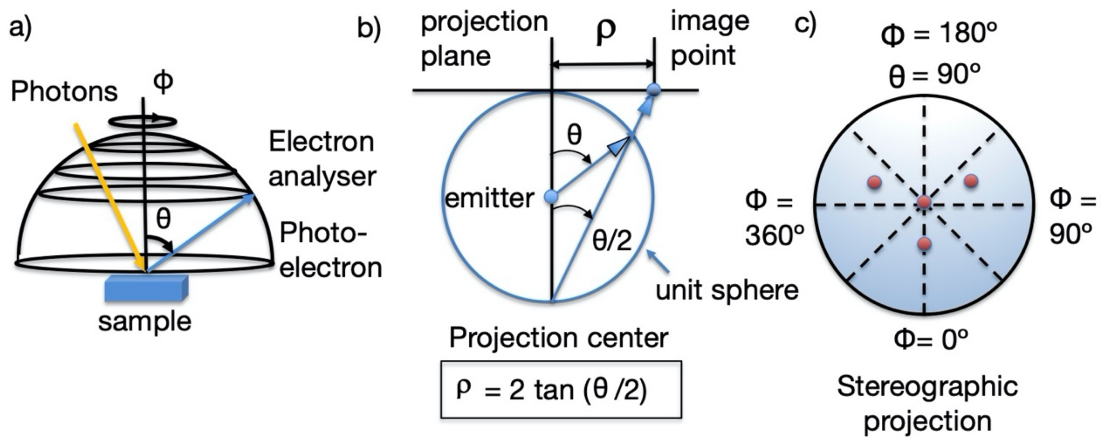

Figure S3: Experimental configuration and full hemispherical (2π) angular intensity modulation patterns representation. Panel (a) shows a typical experimental angle-resolved photoemission experiment. The polar and azimuthal angles are automatically scanned and fully synchronised with the acquisition of a selected photoemission spectrum at each angular position. The intensity of the chosen photoelectron peak (at a given kinetic energy) recorded is represented as a function of the polar and azimuthal angles that are stereographically projected, as shown in (b) and (c). The intensity modulations are presented together with the high symmetry direction of the samples in the real space.

Interpretation of the XPD intensity modulation patterns associated with the core-level emission is based on simulations represented by a spherical electron wave emanating from the emitter atom in the crystal structure. Subsequent interference of this wave with components scattered by the surrounding atoms of a well-defined cluster, representing the sample structure, lead to the observed diffraction intensity modulation patterns. Simulating single or multiple scattering processes depending on the precision and their different approximation of the calculations, XPD intensity modulation patterns are simulated and compared to the experimental XPD patterns. Further details are given below.

1.2- Photoemission matrix element

As it is well-known, the photoemission signal is affected by both the one-electron removal spectral function and by the photoemission matrix elements. The

photocurrent defined by Fermi's golden rule, indicated in equation (1), can be determined by the product of three factors.

$$I(\omega, k) = \Sigma_i [|\langle \varphi_f(r,k_f) |H_{el}|\varphi_i(r) \rangle|^2 A_i(\omega, k) f(\omega, k)] \qquad \text{equation (1)}$$

Where $|M^k|^2_{f,i} = |\langle f |H_{el}|_i \rangle|^2$ is the photoemission matrix element, $A_i(\omega, k)$ the one-electron removal spectral function, and $f(\omega,k)$ the Fermi–Dirac distribution function. It is important to note that equation (1) is only strictly valid at equilibrium. Note that the photoemission matrix element modulates the intensities of the photoemitted yield due to the interference of these waves with those scattered by the surrounding atoms in the crystal.

1.3- Scattering final state wave function

As it has been extensively described in previously (1) (2), ARPES directly probes the one-particle spectral function Ai (ω, k), allowing one to achieve insight into the underlying physics and a satisfactory interpretation of the spectra. However, a precise quantitative analysis of the intensity of the PES peaks must necessarily model the photoexcitation process properly by taking into account the matrix element involved, the complex modifications of the wave functions resulting from a specific surface termination, and the effects of multiple scattering and finite lifetimes of the initial and final states.

To summarize, the most straightforward picture of photoemission is that it samples the density of occupied states in the crystal directly. Although often very useful and productive, this interpretation needs to be applied with caution (3). Effects like the finite lifetime of the hole left behind in the crystal usually broaden the spectrum. Also, surface effects and the influence of the photon field can vary the PES intensity, (4)(5). significantly. Finally, and most importantly highlighted in the present work, the scattering and attenuation suffered by the excited electrons before escaping from the crystal can determine the intensity modulation of PES patterns. In particular, the intensity after the scattering depends on the atomic scattering factor, inelastic attenuation, geometrical and scattering phase shifts, and a Debye-Waller factor to take account of elevated temperature effects (6), (7).

2.- Multiple scattering cluster calculations

Multiple scattering cluster calculations were performed using the approach of Fritzsche (6) based on a magnetic quantum number expansion. All sums over magnetic quantum numbers converge rapidly and can be truncated after a few terms without loss of accuracy. By explicitly considering the finite energy resolution in the photoelectron diffraction experiment, additional damping factors arise in the theoretical expression that simulate the PES intensity modulations experimentally recorded as the polar and azimuthal angles are varied. These considerations systematically suppress the contribution of long scattering pathways (*8*). Consequently, third and higher-order scattering events can be largely neglected due to their longer path lengths. Fuller details of the computational procedure for the MSC presented in this work have been given elsewhere (*6*), where the attenuation depths due to elastic and inelastic scattering events have been taken from Seah and Dench (*9*).

2.1- Description of the Si(111)7x7 atomic cluster

The adatoms and rest-atoms Si photoelectron photoelectron diffraction spectra were calculated using silicon cluster of more than 7488 atoms, composed of surface layers simulating the dimer-adatom-stacking fault (DAS) model of the Si(111)7x7 reconstruction and a hemisphere of bulk atoms of the cluster, as is indicated in Figure S4.

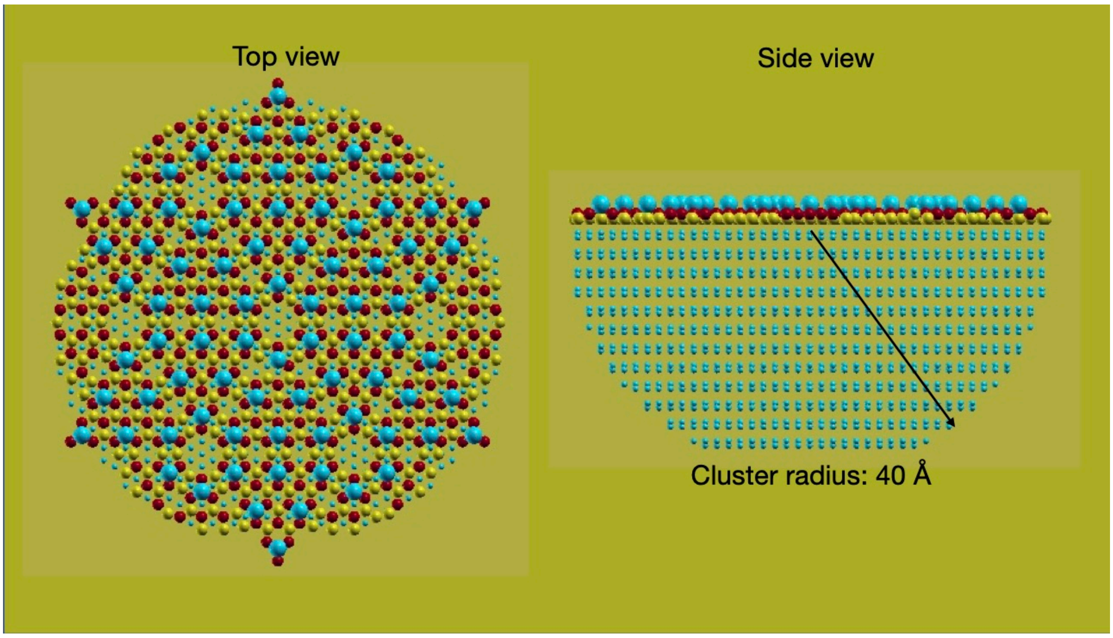

Figure S4. The atomic cluster used for the MSC. Top and side view of the cluster describing the LEED-optimized structure of the Surface region of the cluster and the bulk DAS model with dimer bond length of 2.60 Å. Symmetrised areas of the Si(111)7x7 real-space unit cell are indicated in the left side of the figure, revealing the faulted and unfaulted areas.

As the DAS model is widely accepted and consistent with an extensive set of structural results obtained by analysing many experimental techniques, the atomic coordinates of individual atoms of the surface layers have been introduced in the cluster manually. The atomic positions of the five first near-surface atomic planes (the adatom layer and the two bilayers below) have been optimised by dynamical analysis of the experimental low-energy electron diffraction intensities. This study carried out by Tong et al. (*10*) is consistent with a large body of experimental structural results obtained using scanning tunnelling microscopy, transmission electron diffraction, ion scattering spectroscopy, and x-ray diffraction. The compilation of all these results has allowed individual atomic positions to determine precisely perpendicular distances between atomic layers and lateral relaxations. As is schematically indicated in Figure S4, the MSC cluster used for the simulations presented in this work has 378 atoms in the surface region, which are the atomic positions available in reference (*10*) by Tong et al., and 6700 atoms below, that have the atomic positions of a bulk Si(111) crystal.

3.- References

1. A. Damascelli, Probing the Electronic Structure of Complex Systems by ARPES. *Physica Scripta*. **T109**, 61 (2004).

2. S. Moser, An experimentalist's guide to the matrix element in angle resolved photoemission. *Journal of Electron Spectroscopy and Related Phenomena*. **214**, 29–52 (2017).

3. A. Bansil, M. Lindroos, Importance of Matrix Elements in the ARPES Spectra of BISCO. *Phys. Rev. Lett.* **83**, 5154–5157 (1999).

4. J. B. Pendry, Theory of photoemission. *Surface Science*. **57**, 679–705 (1976).

5. J. F. L. Hopkinson, J. B. Pendry, D. J. Titterington, Calculation of photoemission spectra for surfaces of solids. *Computer Physics Communications*. **19**, 69–92 (1980).

6. V. Fritzsche, A new spherical-wave approximation for photoelectron diffraction, EXAFS and MEED. *J. Phys.: Condens. Matter*. **2**, 1413–1424 (1990).

7. D. P. Woodruff, Photoelectron diffraction: past, present and future. *Journal of Electron Spectroscopy and Related Phenomena*. **126**, 55–65 (2002).

8. V. Fritzsche, Consequences of a finite energy resolution for photoelectron diffraction spectra. *Surface Science*. **265**, 187–195 (1992).

9. M. P. Seah, W. A. Dench, Quantitative electron spectroscopy of surfaces: A standard data base for electron inelastic mean free paths in solids. *Surf. Interface Anal.* **1**, 2–11 (1979).

10. S. Y. Tong, H. Huang, C. M. Wei, W. E. Packard, F. K. Men, G. Glander, M. B. Webb, Low-energy electron diffraction analysis of the Si(111)7×7 structure. *Journal of Vacuum Science & Technology A: Vacuum, Surfaces, and Films*. **6**, 615–624 (1988).
